# A voltammetric sensor based on mixed proton-electron conducting composite including metal-organic framework JUK-2 for determination of citalopram

**DOI:** 10.1007/s00604-021-04835-9

**Published:** 2021-05-11

**Authors:** Maria Madej, Dariusz Matoga, Klaudia Skaźnik, Radosław Porada, Bogusław Baś, Jolanta Kochana

**Affiliations:** 1 Faculty of Chemistry, Department of Analytical Chemistry, Jagiellonian University, Gronostajowa 2, 30-387 Kraków, Poland; 2 Faculty of Chemistry, Department of Inorganic Chemistry, Jagiellonian University, Gronostajowa 2, 30-387 Kraków, Poland; 3 Faculty of Materials and Ceramics, Department of Analytical Chemistry, AGH University of Science and Technology, A. Mickiewicza 30, 30-059 Kraków, Poland

**Keywords:** Citalopram, Metal-organic framework, Electrochemical sensor, Mixed ion-electron conductor, Multi-walled carbon nanotubes, Gold nanoparticles

## Abstract

A voltammetric sensor has been developed based on glassy carbon electrode (GCE) modification with nanocomposite consisting of manganese-based metal-organic framework (JUK-2), multi-walled carbon nanotubes (MWCNTs), and gold nanoparticles (AuNPs) for detection of citalopram (CIT). The composition and morphology of JUK-2-MWCNTs-AuNPs/GCE were characterized by X-ray powder diffraction (XRD), Fourier-transform infrared spectroscopy (FT-IR), energy dispersion spectroscopy (EDS), and scanning electron microscopy (SEM). The electrochemical properties investigated using electrochemical impedance spectroscopy (EIS) and cyclic voltammetry (CV) indicated that the fabricated hybrid material exhibits the properties of mixed ion-electron conductor (MIEC). Using staircase voltammetry (SCV), under optimized conditions, the fabricated sensor shows a linear response in three CIT concentration ranges, 0.05–1.0, 1.0–10.0 and 15.0–115.0 μmol L^−1^, with a detection limit of 0.011 μmol L^−1^. The JUK-2-MWCNTs-AuNPs/GCE was successfully employed for the determination of CIT in pharmaceutical, environmental waters, and biological samples with satisfactory recoveries (98.6–104.8%).

**Supplementary Information:**

The online version contains supplementary material available at 10.1007/s00604-021-04835-9.

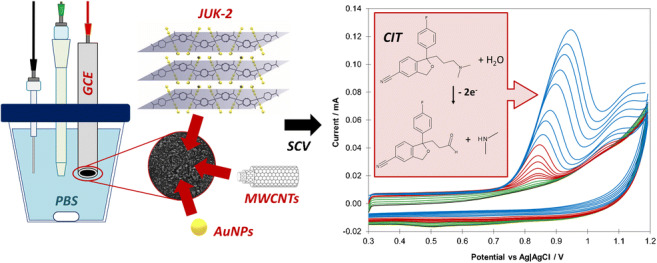

## Introduction

Antidepressants are a group of compounds worthy of attention, due to a constant increase in their consumption in most well-developed countries [1] and hence rising environmental pollution caused by these active substances and their metabolites. One of the most prescribed antidepressants belongs to selective serotonin reuptake inhibitors (SSRI), whose mode of action is based on blocking the uptake of serotonin, thus inducing the neurotransmission between neurons. Citalopram (CIT), as the SSRIs representative, occurs in commercially available formulations as a racemic mixture, but its pharmacological effect is related to the S-enantiomer. CIT is metabolized mainly in the liver via oxidation and excreted in urine, with approx. 20% in unmetabolized form [2, 3]. Numerous studies prove the presence of antidepressants, especially citalopram, venlafaxine, sertraline, and fluoxetine, in various elements of the environment, such as surface water, municipal sewage, or bottom sediments at the concentration level reaching μg L^−1^ [4, 5]. Due to the relatively high persistence in the aquatic environment, even such small amounts of psychoactive substances pose a risk of bioaccumulation in aquatic organisms and may affect their basic life functions [6]. As a result, many analytical methods have been introduced to determine CIT in tablets, water, and biological samples, most notably chromatographic [7] and capillary electrophoretic [8]. Also, a few works based on voltammetric techniques have been reported for CIT determination using bulk glassy carbon electrode [9] or electrodes modified with carbon nanostructures, metal or metal oxide nanoparticles, as well as conducting polymers [10–13].

Metal-organic frameworks (MOFs) are relatively new class of materials, which belong to a wide group of porous coordination polymers with unique, two- or three-dimensional structures [14]. Their excellent physicochemical properties, such as high surface area (up to 7800 m^2^ g^−1^ [15]), high porosity (with pore volumes up to 93% of the total volume of the material [16]), flexibility, and good thermal stability, made MOFs useful as drug carriers, heterogeneous catalysts, materials used for gas storage and separation of hazardous compounds [14]. Because of these attractive features, metal-organic frameworks have been also explored for the development of sensors providing a very good alternative to carbon materials, molecularly imprinted polymers, and zeolites commonly used in this field [17, 18].

A significant limitation of using MOFs to construct electrochemical sensors is that most of these materials are electrically insulating, as composed of hard metal centers and redox-inactive organic linkers with hard donor atoms that cannot provide efficient electron transfer pathways. The improvement of electric conductivity properties of MOF-based sensors can be achieved through a variety of strategies, including incorporation of guest molecules, doping with redox-active compounds (like ferrocene, iodine or metallic species), as well as hybridization with materials exhibiting high electron conductivity, like carbon nanostructures or polymers [19]. On the other hand, conductive MOFs have recently been developed as proton-conducting materials [20], which can also be used for the design of nanostructured electrodes and sensing applications. Finally, in general, mixed ionic-electronic conductors (MIECs), capable of conducting both electrons and ions, are materials that can serve a unique function for electrochemical devices including sensors [21]. Since pristine MIEC materials are rare, they typically come in blended architectures, composed of two types of conductors such as the PEDOT/PSS composite, used for sensitive sensing of glucose and other biomolecules from saliva [22]. To our knowledge, however, literature examples of using MOF-based MIEC composites for electrochemical sensing are lacking.

In this work, we demonstrate using a proton-conducting MOF for the preparation of a MIEC composite for electrochemical sensing application. For this purpose, we selected {(NH_4_)_2_[Mn(ina)_2_(NCS)_2_]}_n_·xH_2_O (JUK-2; Hina = isonicotinic acid) which exhibits two-dimensional layered microporous structure with interlayer separation of 9.63 Å, high proton conductivity above 4 × 10^−4^ S cm^−1^ (in the 70–90% relative humidity range at 25 °C), and good stability in aqueous media [23]. Interestingly, JUK-2 can only be obtained by a solvent-free mechanochemical method, and it is achievable neither by conventional solvothermal methods nor by immersion in solutions. Generally, in contrast to costly and non-ecological syntheses in solution, a solvent-free mechanochemistry has increasingly become a green alternative for the preparation of various materials including MOFs [24]. The mechanochemical preparation of JUK-2 relies on grinding a non-conducting 2D MOF precursor, {[Mn_2_(ina)_4_(H_2_O)_2_]·2EtOH}_n_ (JUK-1) [25] with ammonium thiocyanate. This solid-state reaction involves the NCS^−^ complexation, a significant rearrangement of intralayer coordination bonds (unzipping of JUK-1 bilayers to JUK-2 monolayers) and introducing of extraframework NH_4_^+^ ions as proton carriers [26].

The main aim of this work was to develop a simple voltammetric sensor employing the manganese-based metal-organic framework (JUK-2) for CIT determination. The electrode composite, consisting of layered proton-conducting JUK-2 and nanomaterials exhibiting good electrical properties (multi-walled carbon nanotubes (MWCNTs) and gold nanoparticles (AuNPs)), demonstrated mixed proton-electron conductivity. Optimization of electrode-modifying mixture composition and measurement conditions allowed to develop a sensor exhibited good analytical parameters: high sensitivity, wide linear ranges and low detection limit. The usefulness of proposed JUK-2-MWCNTs-AuNPs/GCE was confirmed by CIT quantification in pharmaceutical, environmental waters, and biological samples.

To our knowledge, the application of MOF-based sensor for citalopram determination, as well as for any other antidepressant, has not been reported in literature so far.

## Experimental

### Apparatus

For voltammetric measurements, standard three-electrode system, consisting of Pt wire as the auxiliary electrode, silver chloride electrode (Ag/AgCl, 3 M KCl, Mineral, Poland) as a reference electrode, and bare or modified glassy carbon electrode (GCE, φ = 3 mm, BASi, USA) as the working electrode, was utilized and controlled by the M161 multipurpose electrochemical analyzer combined with M164 electrode stand (both mtm-anko, Poland) with measurement accuracy of ±1 mV for potential and ± 0.1 nA for current. The impedance spectra were recorded with the use of μAUTOLAB III analyzer (EcoChemie, the Netherlands) with NOVA 2.0 software. The phase composition of optimal hybrid material was verified by measuring XRD patterns on a Rigaku Miniflex 600 diffractometer with Cu-Kα radiation (*λ* = 1*.*5418 Å) in a 2θ range from 3° to 80° with a 0.05° step and 0.5° min^−1^ scan speed. Scanning electron microscope Tescan Vega3 (ThermoFisher Scientific, USA) with energy-dispersive X-ray spectroscopy EDS attachment was used for the surface and elemental analysis of GCE covered with final nanocomposite. FTIR spectra were recorded on a Thermo Fisher Scientific Nicolet iS10 FT-IR spectrophotometer equipped with an iD7 diamond ATR attachment.

### Reagents and materials

The JUK-2 MOF was synthesized by a mechanochemical method according to the procedure described in [23]. Multi-walled carbon nanotubes (length: 0.1–10 μm; internal diameter: 2–6 nm) were purchased from Sigma-Aldrich (USA). The gold nanoparticle (AuNPs) suspension (average diameter ~ 80 nm; 4.8 × 10^9^ particles mL^−1^) was prepared by tetrachloroauric acid reduction with citric acid, according to the procedure described in [27]. The CIT standard stock solution (1.0 mmol L^−1^) was obtained by dissolving of an appropriate amount of citalopram hydrobromide (LGC, Great Britain) in water and then stored in the fridge. Acetic acid, ascorbic acid, disodium hydrogen phosphate, potassium chloride, potassium hexacyanoferrate(II), potassium hexacyanoferrate(III), and sodium dihydrogen phosphate were obtained from Chempur (Poland). Ammonia, ammonium chloride, and sodium acetate were obtained from Merck (Germany). The phosphate, acetate, and ammonium buffer solutions were prepared as described in [9]. Glucose (Micropharm, Poland), hexadecyltrimethylammonium bromide, lactose, magnesium stearate, sodium dodecyl sulfate, starch, talc, titanium dioxide, and Triton X-100 (Sigma-Aldrich, USA) were used for interference studies. Medicaments containing citalopram hydrobromide Oropram (Pluspharma, Austria) and Cital (Glenmark, India) were purchased in local pharmacy. Certified reference materials (CRMs) of synthetic urine Seronorm Trace Elements Urine (Sero, Norway), synthetic serum Nortrol (Thermo Scientific, Finland), Surface Water (SPS–SW1 Batch no. 114), and Waste Water (SPS–WW2 Batch no. 113) (Spectrapure Standards AS, Norway) were used as samples with complex matrix. For that purpose, also water sample from Vistula River was collected in Krakow. All aqueous solutions were prepared with chemicals of analytical grade purity and distilled water from the HLP 5 system (Hydrolab, Poland).

### Standard procedures

#### Metal-organic framework-based sensors fabrication

Due to the high stability of JUK-2 in an aqueous environment, all suspensions used for GCE surface modification were prepared with distilled water as a solvent. For that purpose, one-component suspensions containing 4 mg mL^−1^ of JUK-2 and 1 mg mL^−1^ of MWCNTs were prepared by weighting appropriate amounts of each nanomaterial, mixing them with 2 mL of distilled water and then homogenized with the use of ultrasonic bath for 15 min. For the preparation of the composite consisting of both JUK-2 and MWCNTs, carbon nanotubes were mixed with distilled water and sonicated with an ultrasonic bath for 15 min. After that, the appropriate volume of the obtained MWCNTs suspension was transferred to the Eppendorf containing JUK-2 and sonicated again for 15 min. In the case of the final nanocomposite, an appropriate volume of AuNP suspension was added to the suspension consisting of JUK-2 and MWCNTs and homogenized using vortex for 5 min. All tested suspensions were stored in room temperature without access to light.

The GC electrode modification was preceded by polishing its surface on a polishing cloth (Leco, USA) with 0.05-μm alumina suspension (Buechler, USA) for 30 s and rinsing with strong stream of distilled water. Cleaned GCE was air dried, and then 5.0 μL of tested one- or multi-component suspension, previously homogenized for 5 min using vortex, was applied on the surface of the GCE and left to dry at room temperature.

#### Procedure of measurements

The performance of bare GCE, JUK-2/GCE, MWCNTs/GCE, JUK-2-MWCNTs/GCE, and JUK-2-MWCNTs-AuNPs/GCE was verified on the basis of cyclic voltammetry measurements conducted for [Fe(CN)_6_]^3-\4-^ reversible redox system in 0.1 mol L^−1^ KCl, with potential range between −0.4 and 0.8 V, using the scan rate of 0.05 V s^−1^. The CV experiments were also performed for CIT in 0.1 mol L^−1^ phosphate buffer solution (PBS) pH 7 in the potential range 0.3–1.2 V, scan rate 0.1 V s^−1^, accumulation time (t_acc_) of 60 s, and accumulation potential (E_acc_) of 0 V. The electrochemical behavior of CIT on JUK-2-MWCNTs-AuNPs/GCE was examined under the same conditions.

The impedance spectra were recorded in the frequency range 100 kHz – 25 mHz, using sinusoidal signals with an amplitude of 10 mV imposed on the formal potential of the redox pair [Fe(CN)_6_]^3−/4-^ at concentration of 0.2 mmol L^−1^ in 0.1 mol L^−1^ KCl solution. To obtain the key figures of merit, the equivalent electrical circuit was fitted to every recorded impedance spectrum.

For quantitative determination of CIT, staircase voltammetry in both anodic and cathodic direction from 0.3 to 1.2 V was applied, with potential step (E_s_) 8 mV and step width (t_s_) 80 ms (60 ms of waiting time and 20 ms of current sampling time), which corresponded to the scan rate of 0.1 V s^−1^. Before each measurement, E_acc_ of 0 mV for 180 s was applied to the working electrode. For each CIT concentration, three voltammograms were recorded, and for data analysis, the mean value of peak current was taken into consideration.

#### Samples preparation

The stock solution with expected CIT concentration of 1.250 mmol L^−1^ for each commercial medicament was prepared according to the procedure described in previous work [9]. For determination of CIT, 40.0 μL of selected stock solution was added to 10 mL of 0.1 mol L^−1^ PBS pH 7, and then measurements were conducted according to the standard addition calibration method. For each concentration, three subsequent voltammograms were recorded, and after background subtraction, the mean value of achieved peak current values was calculated.

The sample of river water was subjected to filtration and stored in the fridge before analysis. For river water samples and CRMs of surface water and waste water, SCV measurements were performed, and no signals indicating the presence of the analyte in the samples were observed. Thus, they were enriched with CIT, to obtain concentrations of 5.0 μmol L^−1^, diluted twice with the supporting electrolyte and analyzed analogously as pharmaceutical samples.

The synthetic urine and blood CRMs were spiked with CIT in order to obtain concentration of 100.0 and 400.0 μmol L^−1^, respectively. For SCV determination, 50 μL of spiked urine or 12.5 μL of spiked serum was added to the 0.1 mol L^−1^ PBS pH 7, and then measurements were conducted as described for previous samples.

## Results and discussion

### Choice of materials

At the stage of an electrochemical sensor constructing, the selection of materials used for electrode modification plays a crucial role for achieving high sensitivity and stability of its performance. Since the goal of our research was to develop a MOF-based sensor, various MOFs were first investigated with regard to its stability in aqueous media, durability on the GCE surface, and its electrochemical performance in both model redox probe [Fe(CN)_6_]^3−/4-^ and CIT solution. Out of several MOFs, with different active centers (e.g., Mn^2+^, Cd^2+^, Zn^2+^) as well as various organic linkers, JUK-2 exhibited the best performance, due to its proton-conductive properties, high stability in the aquatic media, and ability to form a highly homogeneous layer on the GCE surface. Since the JUK-2 MOF itself shows neither electron conductivity nor electrocatalytic properties, it was combined with materials that exhibit such features. For this purpose, components like mesoporous carbon CMK-3, MWCNTs, Nafion®, and AuNPs were investigated. From all the tested materials, MWCNTs and AuNPs gave the best results, exhibiting extraordinary properties of a hybrid composite, which were not observed with the other tested materials. Thus, they were used in further investigations. Additionally, various methods of materials deposition onto the GCE surface were tested, including the deposition of a composite from one suspension containing several materials or the deposition of materials separately from single-component suspensions. Accordingly, the deposition of a composite from multi-component suspension seemed to be the best way for sensor preparation.

### Electrochemistry of studied modified electrodes

**Fig. 1:**
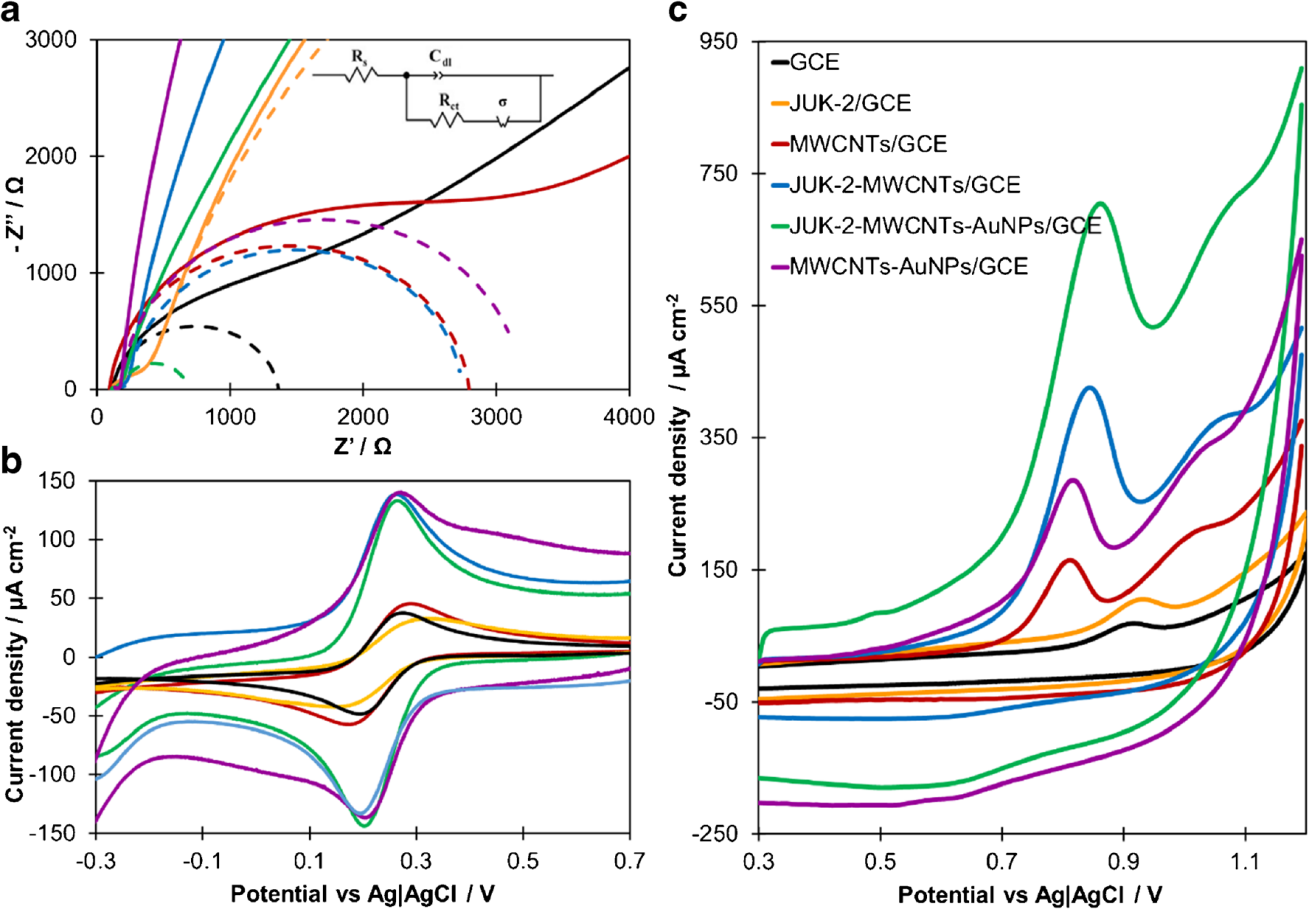
The impedance spectra (**a**) recorded for [Fe(CN)_6_]^3-\4-^ (0.2 mmol L^−1^) in 0.1 mol L^−1^ KCl solution and corresponding cyclic voltammograms (**b**) (scan rate 0.05 V s^−1^); (**c**) CV curves recorded for 0.1 mmol L^−1^ of CIT in PBS (pH 7, 0.1 mmol L^−1^) with scan rate 0.1 V s^−1^, E_acc_ = 0 mV, and t_acc_ = 60 s. The electrodes were marked as follows: GCE, black line; JUK-2/GCE, orange line; MWCNTs/GCE, red line; JUK-2-MWCNTs/GCE, blue line; MWCNTs-AuNPs/GCE, violet line; JUK-2-MWCNTs-AuNPs/GCE, green line

The electrochemical impedance spectroscopy (EIS) and cyclic voltammetry (CV) were used to describe the surface modification of GCE with selected nanomaterials, namely, JUK-2, MWCNTs, and AuNPs. In order to investigate the influence of particular nanocomponents on the metrological parameters of the working electrode, the Nyquist plots for bare GCE, JUK-2/GCE, MWCNTs/GCE, JUK-2-MWCNTs/GCE, MWCNTs-AuNPs/GCE, and JUK-2-MWCNTs-AuNPs/GCE in 0.1 mol L^- 1^ KCl solution containing 0.2 mmol L^−1^ [Fe(CN)_6_]^3−/4-^ were recorded (Fig. 1a).

Based on the obtained EIS spectra, the characteristic parameters such as the charge transfer resistance (R_ct_), Warburg impedance modulus (σ), differential capacity of the double layer (C_dl_), as well as heterogeneous rate constant of the electrode reaction (k_s_) can be determined. For the impedance spectra recorded at the formal potential of a given depolarizer, the charge-transfer resistance and Warburg impedance modulus are expressed by the following formulas [28]:

1 $$ {R}_{ct}=\frac{RT}{n^2{F}^2{A}_{eff}{k}_s}\cdotp \frac{2{\left({D}_O{D}_R\right)}^{1/4}}{D_O^{1/2}{C}_O+{D}_R^{1/2}{C}_R} $$

2 $$ \sigma =\frac{RT}{n^2{F}^2{A}_{eff}\sqrt{2}}\cdotp \frac{4}{D_O^{1/2}{C}_O+{D}_R^{1/2}{C}_R} $$

where Ris the universal gas constant (R = 8.314 J mol^−1^ K^−1^), T is the temperature, n is the number of the exchanged electrons, F is the Faraday constant (F = 96,485.3 C mol^−1^), A_eff_ is the effective surface area, k_s_is the heterogenic rate constant, D is the diffusion coefficient and c is the concentration of the reduced (*c*_*R*_) and oxidized form (*c*_*O*_).

By dividing these equations, the expression for the heterogenic rate constant k_s_ can be obtained:

3 $$ {k}_s=\frac{\sigma }{R_{ct}}\cdotp \frac{{\left({D}_O{D}_R\right)}^{1/4}}{\sqrt{2}} $$

According to literature, diffusion coefficients D_O_ and D_R_ for the [Fe(CN)_6_]^4−/3-^ redox system are equal 7.2 × 10^−6^ cm^2^ s^−1^ [29]. EIS characteristic parameters determined based on the above-mentioned equations were summarized in Table 1.

**Table 1:** The comparison of tested electrodes based on EIS measurements (*n* = 3)

Electrode	*R*_*ct*_/kΩ	*σ*/kΩ s^-1/2^	*C*_*dl*_/μF cm^−2^	*k*_*s*_/× 10^−5^ m s^−1^
GCE	1.25 ± 0.05	9.6 ± 0.2	11.5 ± 0.3	(14.6 ± 1.0)
JUK-2/GCE	14.7 ± 0.3	10.5 ± 0.1	278 ± 18	(1.30 ± 0.08)
MWCNTs/GCE	2.73 ± 0.15	8.8 ± 0.1	10.5 ± 0.7	(6.18 ± 0.06)
JUK-2-MWCNTs/GCE	2.50 ± 0.05	10.4 ± 0.2	2220 ± 40	(7.89 ± 0.05)
MWCNTs-AuNPs/GCE	3.00 ± 0.50	11.1 ± 0.4	5480 ± 80	(6.96 ± 0.05)
JUK-2-MWCNTs-AuNPs/GCE	0.50 ± 0.01	9.0 ± 0.1	1030 ± 25	(34.2 ± 0.8)

The relatively small dispersion of σ, as well as characteristic, asymmetric shape of redox peaks for model probe [Fe(CN)_6_]^3−/4-^ observed on CV curves, indicate that for all tested electrodes, mass transfer is controlled by linear diffusion. The value of C_dl_ for GCE is typical for 0.1 mol L^−1^ KCl, which proves that no additional adsorption occurs at bare electrode. The recorded EIS spectra show that the modification of GCE surface with JUK-2 contributes to a distinct increase of R_ct_ and a decrease of rate constant k_s_, which proves that the employed metal-organic framework hinders electron transfer due to its poor electron conductivity. Moreover, JUK-2 causes a significant increase in C_dl_, which is a highly undesirable effect. The modification of GCE with MWCNTs results in a slight increase of R_ct_ and a decrease of k_s_, but to a much lesser extent than in the case of JUK-2. Compared with the JUK-2/GCE, the electrode modified with both JUK-2 and MWCNTs is characterized by much better metrological parameters, thus confirming the legitimacy of using JUK-2 as an electrode modifier. However, such a two-component modifying layer causes a significant increase in the C_dl_, and consequently the growth of capacitive current, which is the main component of the background current in voltammetric techniques. The introduction of AuNPs into the modifying layer lowers the C_dl_ value twice, which is undoubtedly a favorable phenomenon. In addition, the electrode prepared in this way has the lowest R_ct_ value (approx. 2.5 times lower compared to the unmodified GCE), which is also reflected in a high value of the constant k_s_. Meanwhile, the MWCNTs-AuNPs/GCE exhibits a very high C_dl_ value and a moderate k_s_, lower than for JUK-2-MWCNTs/GCE and JUK-2/MWCNTs-AuNPs/GCE, which confirms the crucial role of JUK-2 in the constructed sensor. The obtained results undoubtedly prove that the combination of JUK-2 with MWCNTs and AuNPs improves the metrological parameters and electrical properties of the bare GCE and provides the most favorable signal-to-noise (S/N) ratio.

In order to confirm the electrode behavior observed in EIS measurements, CVs in 0.1 mol L^−1^ KCl containing 1.0 mmol L^−1^ of [Fe(CN)_6_]^3−/4-^ were recorded (see Fig. 1b). Based on Randles-Ševčík equation (Eq. 4), the electrochemical active surface area of each tested electrode was calculated [30, 31]:

4 $$ {I}_p=\left(2.69\cdot {10}^5\right){n}^{3/2}{A}_{el}{D}^{1/2}{v}^{1/2}c $$

where I_p_ is the peak current, A_el_ the electrochemical active surface area of electrode, and ***v*** the scan rate (***v*** = 0.05 V s^−1^), D = 7.2 **×** 10^−6^ cm^2^ s^−1^ [29].

A well-defined voltammogram with symmetric anodic and cathodic peaks and the peak separation (ΔE) of 75 mV was observed at the bare GCE. After modification of electrode with JUK-2, the anodic and cathodic current density decreased, with a growth of ΔE to 160 mV, which is justified by the low electrical conductivity of JUK-2 and the resulting significant increase in the values of R_ct_ and C_dl_ (Table 1). The deposition of MWCNTs on the GCE caused a distinct growth of R_ct_ due to the weak physical adhesion of MWCNTs to the electrode surface. Also, a slight increase of the modulus σ and capacity C_dl_, as well as an almost twofold decrease of rate constant k_s_ in reference to bare GCE was observed. As a result, for MWCNTs/GCE, a slight increase of redox peaks and a growth of ΔE to 116 mV, compared with bare electrode, was noticed. The JUK-2-MWCNTs/GCE showed a distinct enhancement of the anodic and cathodic peak current density (above 3 times compared with the bare GCE, JUK-2/GCE and MWCNTs/GCE). However, the high value of the differential capacity C_dl_ (almost 100 times higher than for the GCE) makes the S/N ratio unfavorable and the capacitive current is not compensated. The best electrical properties from all considered sensors ensure the JUK-2-MWCNTs-AuNPs/GCE. For this electrode, the lowest R_ct_ value and the highest value of the rate constant reaction k_s_ were obtained; hence the best shaped voltammograms and the most favorable S/N ratio were achieved.

The calculated electrochemical active surface area of JUK-2-MWCNTs-AuNPs/GCE (0.19 cm^2^) is comparable to JUK-2-MWCNTs/GCE (0.20 cm^2^), ca. 50% higher than for MWCNTs-AuNPs/GCE (0.13 cm^2^) and more than twice as large as for MWCNTs/GCE (0.093 cm^2^), JUK-2/GCE (0.079 cm^2^), and bare GCE (0.070 cm^2^). The significant expansion of electrochemical active surface area of JUK-2-MWCNTs-AuNPs/GCE, accompanied by facilitated electron transfer, enables to increase the efficiency of the occurring electrode reaction, which manifests in an outstanding increase of recorded current and thus higher sensitivity.

The performance of tested modified electrodes was investigated by CV in 0.1 mol L^−1^ PBS (pH 7) containing 100 μmol L^−1^ of CIT (Fig. 1c). Before surface modification, the GCE showed a poor, one anodic wave (40.3 μA cm^−2^) at the potential of 0.91 V. For JUK-2/GCE, the oxidation peak current density slightly decreased to 38.5 μA cm^−2^ with minor shift of peak position towards positive potentials due to low electric conductivity of the JUK-2. These observations are consistent with the JUK-2/GCE properties determined in EIS measurements. The modification of electrode with MWCNTs resulted in more than twofold increase of recorded anodic current density (106.1 μA cm^−2^) accompanied by a shift of the peak potential in the cathodic direction up to 0.82 V. Such a large shift of the CIT oxidation peak indicates a significant reduction of the activation energy of the drug electrooxidation process, and thus the creation of many new active centers on the sensor surface. This favorable effect is amplified by the decoration of MWCNTs with Au nanoparticles. However, both electrodes, i.e., MWCNTs/GCE and MWCNTs-AuNPs/GCE, are characterized by poor reproducibility and low mechanical durability. Further improvement of the sensor performance was achieved by GCE modification with JUK-2-MWCNT composite. The observed favorable enhancement of the signal at JUK2-MWCNTs/GCE is almost twice higher than the predicted growth resulting from the combination of individual components properties, which indicates the presence of synergistic effect of JUK-2 and MWCNTs. Among all, the best electrochemical and functional parameters were obtained for JUK2-MWCNTs-AuNPs/GCE, which showed a tenfold increase of anodic peak current density (402.6 μA cm^-2^) in comparison to bare GCE.

The excellent performance of JUK-2-MWCNTs-AuNPs/GCE towards CIT could be contributed to several factors. The combination of proton conductive JUK-2 crystallites with electron conductive MWCNTs and AuNPs leads to enlargement of the active surface area and formation of a hybrid composite, which acts as mixed ion-electron conductor (MIEC) [32]. Such a feature should be especially favorable for electrochemical processes in which electron transfer is accompanied by deprotonation, as in the case of CIT. This phenomenon could also explain the synergistic effect of JUK-2, MWCNTs, and AuNPs.

### Composition and morphology of the electrode nanocomposite

The amounts of chosen components, JUK-2, MWCNTs, and AuNPs, played a significant role in the electrochemical performance of a modified electrode. Therefore, different mass ratios of JUK-2 and MWCNTs in composite suspensions (from 1:3 to 9:2) were evaluated on the basis of CV measurements conducted in 0.1 mol L^−1^ KCl for increasing concentration of [Fe(CN)_6_]^3−/4-^ (Fig. S1a). It was observed that the sensitivity of modified electrodes to the model redox probe was increasing with a growing amount of JUK-2 relative to MWCNTs, reached a maximum for mass ratio of 4:1 and then slightly decreased. As a result, the optimal suspension containing 4 mg mL^−1^ of the JUK-2 and 1 mg mL^−1^ of MWCNTs was selected for subsequent studies.

After introduction of AuNPs (5% vol.) to the electrode nanocomposite, the sensitivity of measurements conducted for the model redox probe fell by about 12% and it remained constant regardless of the amount of AuNPs. Hence, the measurements in the presence of target analyte CIT were performed. For that purpose, CV in 0.1 mol L^−1^ PBS (pH 7.0) for increasing concentration of CIT were recorded (Fig. S1b). It is clearly visible that sensitivity for CIT determination is strongly affected by the amount of AuNPs. The highest sensitivity was obtained using electrode modified with nanocomposite containing 20% AuNPs by volume. In comparison to JUK-2-MWCNTs/GCE, the addition of AuNPs resulted in 3.5 times improvement of sensitivity. Thus, the optimal nanocomposite consisted of 4 mg mL^−1^ of JUK-2, 1 mg mL^−1^ of MWCNTs and 20% vol. of AuNPs. No influence of AuNPs on the sensitivity of measurements conducted in the presence of Fe^2+^/Fe^3+^ in comparison to significant increase of sensitivity observed for CIT can be related to different reaction mechanisms. The CIT oxidation is controlled by adsorption, and in this case, AuNPs could act as active centers on which CIT molecules easily adsorb, which distinctly improve the sensitivity. Whereas for Fe[(CN)_6_]^3−/4-^ both oxidation and reduction processes are controlled by diffusion, so electrode reactions do not run directly on the electrode surface; therefore the presence of AuNPs has no impact on their course.

**Fig. 2:**
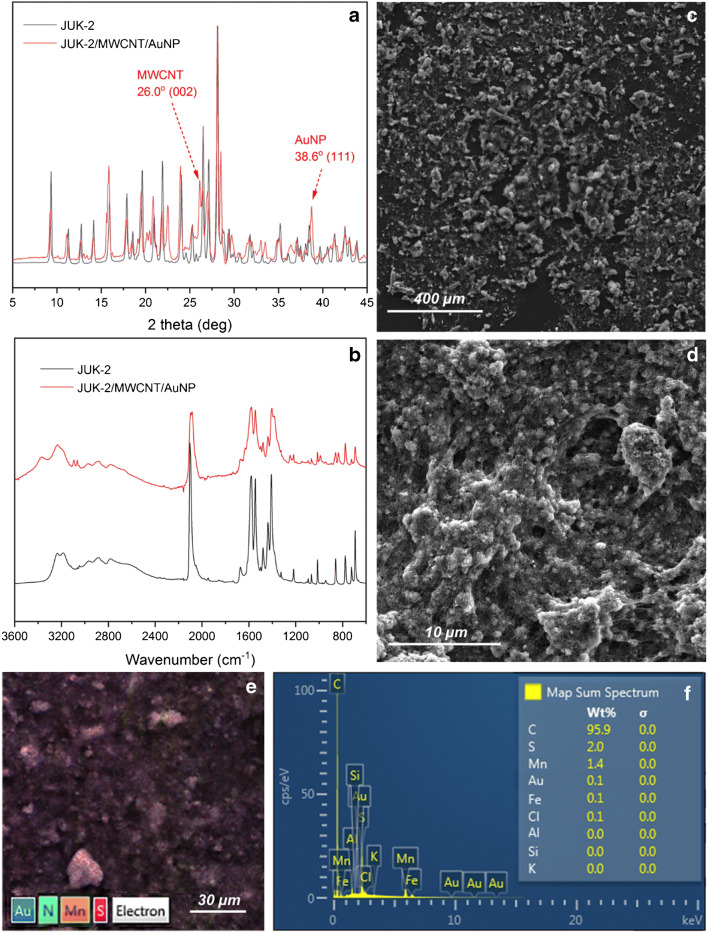
XRD patterns (**a**) and FT-IR spectra (**b**) for pristine JUK-2 (black) and the JUK-2-MWCNTs-AuNPs composite (red); SEM images of glassy carbon electrode surface modified with final nanocomposite layer (**c**, **d**) with corresponding EDS mapping (**e**) and elemental composition estimated by EDS analysis (**f**)

The morphology of the final nanocomposite was specified using powder X-ray diffraction (XRD), scanning electron microscopy (SEM), and energy dispersive spectroscopy (EDS). The XRD patterns of pristine JUK-2 and the JUK-2/MWCNTs/AuNPs composite clearly demonstrate the coexistence of three components in the hybrid material (Fig. 2a). Apart from predominant characteristic peaks of JUK-2 phase in the composite, the extra diffraction peak at 26.0^o^ indicates the presence of MWCNTs with d-spacing of 3.42 Å between graphene sheets. The third component is corroborated by the diffraction peak at 38.6^o^ that corresponds to the (111) plane in the fcc structure of gold nanoparticles. IR spectroscopy reveals that the vibrations of the skeletal atoms remained intact during the composite formation, confirming the retention of the layered JUK-2 structure. Moreover, the presence of a strong CN band at 2100 cm^−1^ proves that thiocyanates remain coordinated as terminal ligands in JUK-2 (Fig. 2b). SEM images (Fig. 2c, d) and EDS elemental mapping (Fig. 2e) of the studied composite additionally confirm the presence of distinct elements (Mn and S, C, and Au) of each of the three phases (Fig. 2f).

In order to estimate the mass fraction of the individual components in the hybrid material, the approximate quantitative analysis of the composite content was investigated, on the basis of AuNP concentration and average nanoparticle size in initial AuNPs suspension, as well as JUK-2 and MWCNTs amount. Performed calculations suggest that optimal nanocomposite contained 80% of JUK-2, 20% of MWCNTs, and 0.1% of AuNPs, respectively. Calculated percentage composition of the optimal nanocomposite is consistent with the results obtained in EDS analysis.

### Investigation of the electrode reaction mechanism

**Fig. 3:**
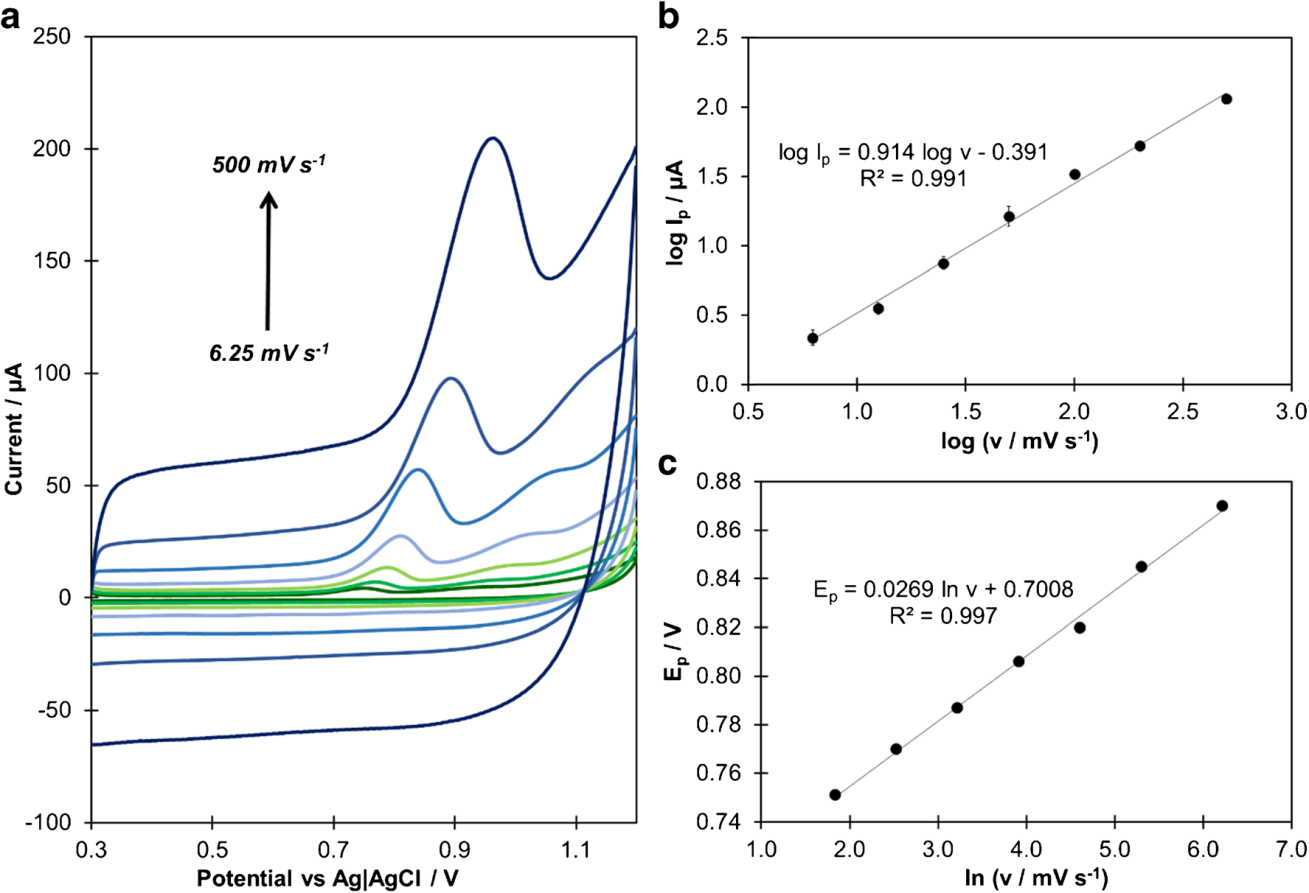
CV curves recorded for scan rate values in the range of 6.25–500 mV s^−1^ (**a**) and their impact on CIT anodic peak current (**b**) and peak potential (**c**) performed in 0.1 mol L^−1^ PBS (pH 7) containing 30 μmol L^−1^ of CIT (E_acc_ = 0 V, t_acc_ = 60 s); *n* = 3

The electrode reactions are governed by either adsorption or diffusion-controlled processes and the systematic analysis of the impact of scan rate (ν) on the recorded peak current (I_p_) can provide evidence to distinguish between these two processes. In order to understand the mechanism of CIT oxidation at JUK-2/MWCNTs-AuNPs/GCE, CV measurements at scan rates ranging from 6.25 to 500 mV s^−1^ were performed (Fig. 3a). In the presence of 30 μmol L^−1^ of CIT, the anodic peak current increased proportionally with scan rate, in accordance with regression equation of I_p_ (μA) = 0.227 ν – 3.852 (R^2^ = 0.992, see Fig. S2). Furthermore, a relationship between logarithm of peak current on the logarithm of scan rate (Fig. 3b) exhibited an excellent linearity (R^2^ = 0.991) with a slope of 0.914, which is close to theoretical value of 1.0, corresponding to adsorption-controlled process [33].

In addition to peak current changes, it can be observed that the oxidation peak potential (E_p_) shifts positively as the scan rate increases (Fig. 3a), which indicates the irreversibility of electrode reaction [34]. According to Laviron equation, for an irreversible, adsorption-controlled process, the dependence between E_p_ and scan rate can be described as follows [35]:

5 $$ {E}_p=A+\frac{RT}{\left(1-\alpha \right) nF}\mathit{\ln}\ v $$

where A is the constant value.

The value of *(1 – α) n* calculated from the slope of E_p_ versus ln *v* dependance (Fig. 3c) was equal to 0.955. For irreversible electrode processes, the charge transfer coefficient (α) is assumed to be 0.5 [35]; hence the number of electrons was estimated as 1.91, which indicates that two electrons are involved in the citalopram oxidation.

**Fig. 4:**
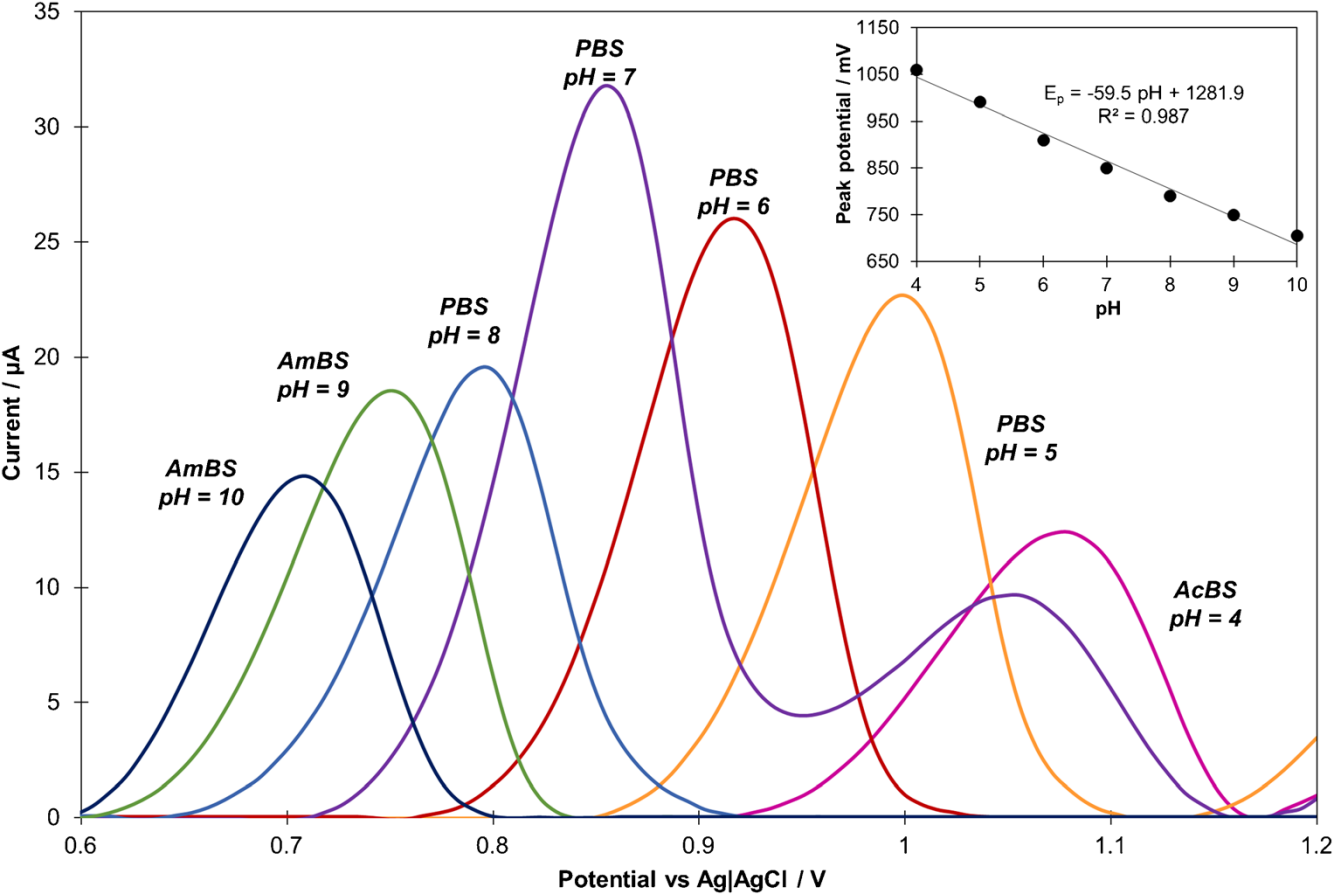
CV curves (subjected to background reduction) recorded in various buffers (a) (each at concentration of 0.1 mol L^−1^) containing 30 μmol L^−1^ of CIT (ν = 0.1 V s^−1^, E_acc_ = 0 mV, t_acc_ = 60 s) and the influence of buffer pH on CIT oxidation peak potential (inset). *AmBS* ammonium buffer solution, *PBS* phosphate buffer solution, *AcBS* acetate buffer solution

In order to investigate the effect of the pH on the oxidation of CIT at the developed sensor, CV were recorded in 0.1 mol L^−1^ acetate (AcBS), phosphate (PBS), and ammonium (AmBS) buffer solutions with pH values ranging from 4.0 to 10.0 (Fig. 4). The obtained voltammograms demonstrate that with increasing pH value, the anodic peak potential shifted linearly in negative direction, indicating the involvement of protons in the electrochemical reaction. The relationship between oxidation peak potential and pH can be described as E_p_ (mV) = −59.5 pH + 1281.9 with R^2^ = 0.987. The achieved directional coefficient is close to theoretical value of 59.0 mV pH ^−1^, suggesting the participation of equal number of protons and electrons in CIT oxidation process [36].

On the basis of obtained results and previous reports concerning the electrooxidation of amine derivatives, it can be concluded that observed anodic peak is related to the irreversible, adsorption-controlled oxidation of tertiary amine group present in CIT molecule through the formation of a cation radical which, after deprotonation and loss of two electrons, transforms into secondary amine and aldehyde [37, 38]. The created amine may undergo further oxidation under appropriate conditions, which may explain the presence of the second, residual anodic wave observed for high concentrations of CIT. These conclusions are consistent with previous reports describing the CIT oxidation at modified electrodes [11, 12]. The proposed mechanism for the electrode reaction is presented in Fig. S3.

### Optimization of experimental parameters for quantitative analysis

The electrochemical processes involving organic compounds are mostly strictly related to the pH of supporting electrolyte. Hence, the influence of pH of different buffers (PBS, AmBS, AcBS) on the oxidation of CIT was investigated not only in terms of the peak position but also its height (Fig. 4). The recorded CV voltammograms demonstrate that the anodic peak current increases rapidly from pH 4.0 to a maximum at pH 7.0, and then it distinctly declines. The observed tendency is consistent with the proposed mechanism of CIT oxidation, which includes proton elimination, so in acidic media the electrode reaction should be obstructed [10]. The dissociation constant (pK_a_) of CIT is close to 9, so below pH 9 CIT molecules are predominantly protonated, which facilitates the course of electrode reaction [11]. With further pH increase, the concentration of the protonated form declines, and thus the peak current also decreases. The above considerations confirm that pH 7 constitutes the best environment for CIT oxidation; therefore all quantitative electrochemical experiments were carried out in PBS with this pH value.

In order to develop a methodology for quantitative analysis of CIT, various voltammetric techniques, such as cyclic voltammetry (CV), staircase voltammetry (SCV), differential pulse voltammetry (DPV), and square wave voltammetry (SWV) were considered. For final procedure, SC voltammetry was selected, since this technique showed a higher sensitivity in comparison to CV and reduced capacitive current, but at the same time it is not a pulse technique such as DPV and SWV, whose application resulted in a drastic, 15-fold decrease in the measured current values, regardless of the used parameters (data not shown). This phenomenon is possibly related with the nature of the electrical double layer that forms at the phase boundary between modified electrode and solution. The optimal conditions for CIT determination using SCV were selected through the examination of the relationship between the recorded oxidation peak current and measurement parameters, such as potential step (E_s_), step width (t_s_), accumulation time (t_acc_) and accumulation potential (E_acc_) (see *Supplementary Material* for details). The anodic peak with satisfactory height and good repeatability was obtained for E_s_ of 8 mV and t_s_ of 80 ms, which corresponds to scan rate equal 0.1 V s^−1^, with t_acc_ equal to 180 s and E_acc_ of 0 mV. Therefore, these parameters were chosen for further studies.

### Analytical performance of the sensor

**Fig. 5:**
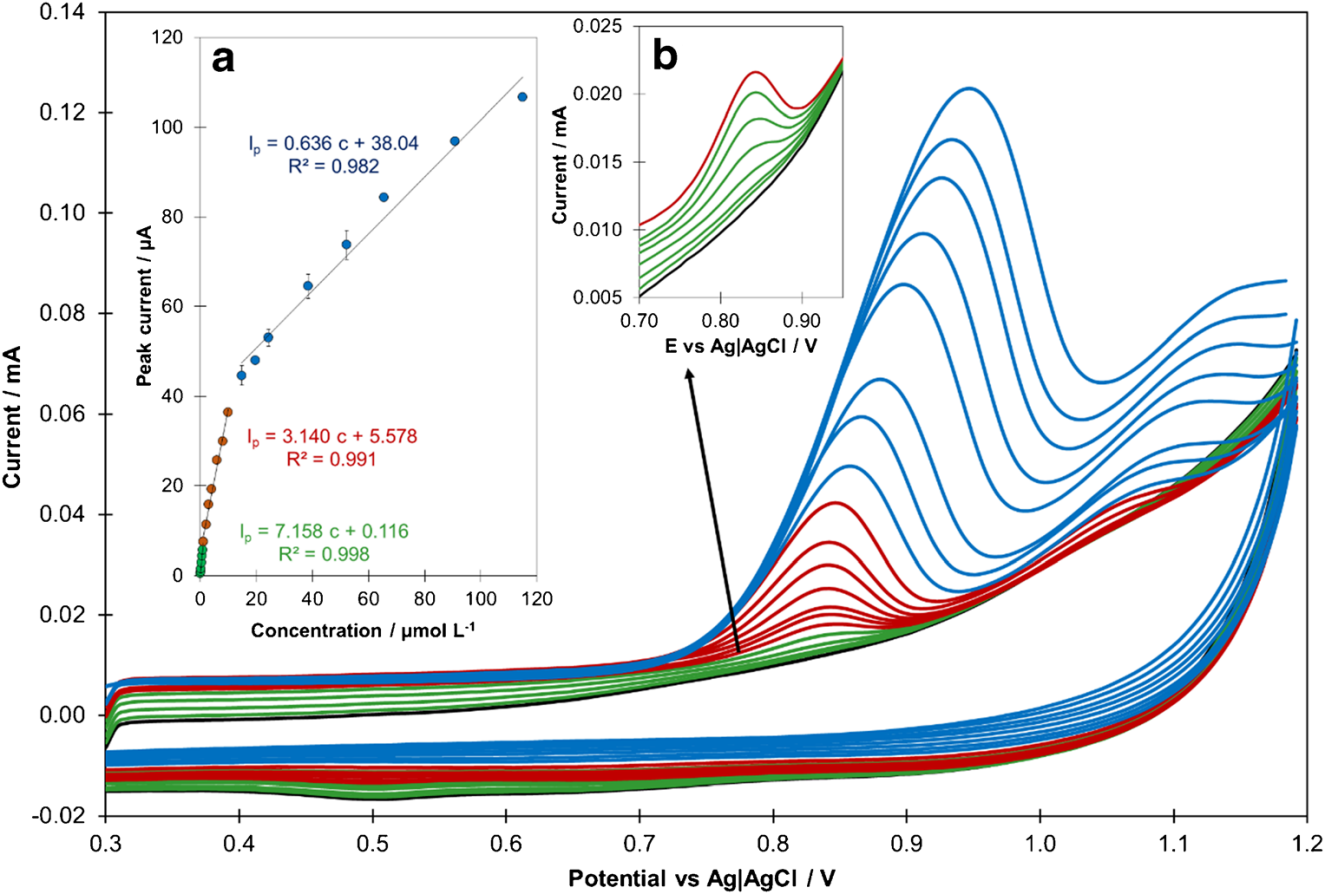
SC voltammograms and corresponding calibration graph (inset a) obtained for different concentrations of CIT in 0.1 mol L^−1^ PBS (pH 7) (black line), *n* = 3; inset b: magnified voltammograms for 0.05–1.0 μmol L^−1^ CIT). SCV parameters: E_s_ = 8 mV, t_s_ = 80 ms, E_acc_ = 0 mV, t_acc_ = 180 s

Under the optimal experimental conditions, the sensitivity of CIT determination in 0.1 mol L^−1^ PBS (pH 7) at JUK-2/MWCNTs-AuNPs/GCE was investigated by SCV (Fig. 5). A good linear relation between the oxidation peak current and the CIT concentration was found. The anodic peak current of CIT increased linearly in three concentration ranges of 0.05–1.0, 1.0–10.0, and 15.0–115.0 μmol L^−1^, with sensitivities of 38.00 ± 0.79 μA L μmol^−1^ cm^−2^, 15.89 ± 0.68 μA L μmol^−1^ cm^−2^, and 3.32 ± 0.47 μA L μmol^−1^ cm^−2^ (mean values for 3 sensors), respectively. The limit of detection (LOD), defined as 3.3 times standard deviation of peak current recorded for 0.05 μmol L^−1^ CIT (*n* = 5) over the calibration slope, was calculated as 0.011 μmol L^−1^, whereas the limit of quantification (LOQ), evaluated as 3LOD, was equal to 0.033 μmol L^−1^ [39].

The reproducibility and stability of the proposed sensor were also examined. The relative standard deviation (RSD) of peak current values recorded in PBS containing 10 μmol L^−1^ of CIT using six independently fabricated electrodes was 2.3%, indicating good reproducibility. Additionally, the short-term stability of the sensor was investigated after storage at air at room temperature for 24 h (Fig. S5). After one day, the recorded signal decreased to almost 50% of the initial peak current, which suggests that the sensor shows its best performance immediately after preparation. Despite the short stability of the sensor, it is noteworthy that the stability of the electrode modifying suspension itself is very high. Even after 6 months, it allows for fabrication of sensors with excellent reproducibility (3.5%). Considering that, the electrode preparation procedure consists of simple and fast one-step modification by drop casting of multi-component suspension onto GCE. The sensor can be easily fabricated just before analysis; thus its short stability does not appear to be a significant limitation.

Comparing to other electrochemical methods developed recently for CIT determination (Table 2), the proposed sensor is characterized by almost the lowest LOD value described in literature so far, with a wide linear range. In contrast to all the modified electrodes presented in Table 2, JUK-2/MWCNTs-AuNPs/GCE is easy to fabricate and does not require the use of harmful solvents, like toluene or DMF. Moreover, at all stages, from synthesis of both JUK-2 [23] and its precursor JUK-1 [25] to CIT quantification, only reagents neutral for environment are used, which promotes the principles of green analytical chemistry (GAC) [40].

**Table 2:** Characteristics of various modified electrodes implemented for CIT determination

Electrode	Technique	Linearity range/μmol *L*^*−1*^	LOD/μmol *L*^*−1*^	Reference
Fe_3_O_4_@[(EtO)_3_Si–L]/MWCNTs/GCE^1^	DPV	0.3–100.0	0.053	[12]
AuPdNPs-GE/AuE^2^	SWV	0.5–50.0	0.049	[13]
MWCNTs-pABSA-β-CD/GCE^3^	DPV	0.09–1.0 1.0–11.0 11.0–100.0	0.044	[11]
ZnO-NPs /MWCNTs/CPE^4^	SWV	0.012–1.54	0.005	[10]
JUK-2-MWCNTs-AuNPs/GCE	**SCV**	**0.05–1.0** **1.0–10.0** **15.0–115.0**	**0.011**	**This work**

^1^Glassy carbon electrode chemically modified with multi-walled carbon nanotubes and core-shell functionalized magnetic nanoparticles of iron(II,III) oxide; ^2^ gold electrode modified with graphene decorated by gold-palladium nanoparticles; ^3^ glassy carbon electrode modified with multi-walled carbon nanotubes coated with poly p-aminobenzene sulfonic acid/β-cyclodextrin film; ^4^ carbon paste electrode modified with zinc oxide nanoparticles and multi-walled carbon nanotubes

### Interference studies

The ability of the fabricated sensor for the determination of CIT in real samples such as environmental water, wastewater samples, and pharmaceuticals was evaluated. For that purpose, a variety of possible interfering organic and inorganic compounds (in concentration between 0.1–100 μmol L^−1^), such as ascorbic acid, glucose, lactose, titanium dioxide, talc, starch, magnesium stearate, Triton X-100 (non-ionic surfactant), sodium dodecyl sulfate (SDS; anionic surfactant), and hexadecyltrimethylammonium bromide (CTAB; cationic surfactant), were tested. The tolerance limit was set as a percentage signal change less than ± 5% corresponding to the peak current recorded for 10 μmol L^−1^ of CIT without and in the presence of an interferent.

The majority of tested interferents, regardless of its concentration, did not affect visibly the recorded signal for CIT (for more details *see* Table S2). Only the presence of magnesium stearate, Triton X-100, and CTAB resulted in a decline of the measured peak current (Fig. S6a), without change of its shape and position. The suppressive influence of magnesium stearate on the recorded signal was observed for its concentration higher than 10 μmol L^−1^. This slight signal drop (less than 12%) is most likely related to the presence of a solid phase in the supporting electrolyte, which inhibits the course of the electrode reaction. This unfavorable effect, which can occur in pharmaceuticals analysis, can be easily eliminated by subjecting samples to filtration. In contrast, both Triton X-100 and CTAB strongly affect the recorded peak current, even at low concentrations of these surfactants. In the case of 10-fold excess of CTAB or Triton X-100 in comparison to CIT concentration, the signal decreases even by 70%. Based on these observations, it can be concluded that the occurrence of cationic or non-ionic surfactants in highly polluted water samples could make the CIT determination difficult; however these surfactants usually occur in environmental waters at a nanomolar level [34, 41]; therefore the risk is relatively low.

The influence of synthetic biological matrices on the recorded signal was verified through testing of increasing percentage content of the Blood Serum or Urine Certified Reference Material (CRM) in PBS volume (Fig. S6b). The obtained results clearly indicate the presence of strong interferences even for small content of biological matrices, which at values of 5% (for urine) and 2% (for serum) lead to complete disappearance of the recorded peak for CIT. However, at low content of biological matrix (not exceeding 0.5% for urine, and 0.1% for serum), the decrease of the signal can be negligible; thus under such conditions, it should be possible to determine CIT, but at relatively high concentrations only (at least 100 μmol L^−1^). In the case of samples with lower CIT concentration, an employment of extraction at a sample preparation stage may be a good alternative for reducing the matrix impact.

The conducted studies indicate that the developed sensor is characterized by good selectivity and can be used for CIT determination in commercial medicaments and water samples without special sample pretreatement. The possible interference risk can be related to the presence of high quantities of non-ionic and cationic surfactants or components of biological matrices. However, these interferences should be compensated by employment of adequate calibration approach, like the standard addition method or more complex approaches, e.g., generalized calibration strategy [42].

### Practical applications of JUK-2-MWCNTs-AuNPs/GCE

To evaluate the analytical applicability and reliability of the JUK-2-MWCNTs-AuNPs/GCE sensor was applied for CIT determination in tablets, surface and waste water CRMs, and synthetic biological samples. Two commercially available pharmaceuticals containing citalopram hydrobromide were analyzed three times, and then the relative error (RE) with confidence intervals (CI) and RSD was calculated (Table 3). For both medicaments the RE value did not exceed 2.5%, and the RSD values were less than 0.5%, indicating good compliance between the obtained value and the amount declared by manufacturer. Thus, it can be concluded that the developed sensor provides a good alternative for real sample analysis of pharmaceutical tablets.

**Table 3:** Determination of CIT in pharmaceutical pills

Sample	**Amount declared by manufacturer/*mg***	**Amount determined/*mg***	Average amount* ±CI/*mg*	RE* /*%*	**RSD** **/*%***
Oropram	20.00	19.57		−2.2	0.45
		19.66	19.57 ± 0.15		
		19.48			
Cital	20.00	19.56		−2.3	0.45
		19.63	19.55 ± 0.14		
		19.46			

*mean value (*n* = 3); *CI* confidence interval calculated for α = 0.05

For Vistula water, Surface water, and Wastewater CRM samples, no target analyte was found; therefore a recovery test was conducted to assess sensor applicability. The obtained results are summarized in Table 4. The reasonable recovery (between 98.6 to 103.8%) with satisfactory RSD (below 1.0%) values suggest that the application of JUK-2-based sensor for CIT determination in environmental samples is characterized by good accuracy and excellent precision.

**Table 4:** Determination of CIT in spiked water and biological samples

**Sample**	**Amount added/**μmol ***L***^***−1***^	**Amount determined/*μmol L***^***−1***^	Recovery* /*%*	**RSD** **/*%***
**Vistula water**	5.00	4.97	98.6 ± 1.4	0.84
		4.93		
		4.89		
**Surface water CRM**	5.00	5.15	103.3 ± 0.7	0.42
		5.19		
		5.15		
**Wastewater CRM**	5.00	5.22	103.8 ± 0.8	0.47
		5.17		
		5.19		
**Synthetic urine**	100.0	99.8	100.2 ± 1.8	1.05
		99.4		
		101.4		
**Synthetic serum**	400.0	417.5	104.8 ± 0.9	0.49
		418.2		
		421.4		

*mean value (n = 3)

The JUK-2-MWCNTs-AuNPs/GCE was also successfully applied for synthetic urine and serum sample analysis. The determination of CIT in biological samples was performed in the same manner as for water analysis, and based on obtained results, the recovery and RSD values were calculated (Table 4). Using the previous interference tests performed for biological matrices (Fig. S6b), it was possible to estimate sample dilution rates, which allowed for interference effect minimization. Thus, the performed analyses are characterized by acceptable precision and accuracy, with recovery rates of 100.2 and 104.8%, with RSD values lower than 1.05%, indicating that the proposed method could be efficiently used for CIT analysis in samples with complex matrix.

## Conclusions

This work describes a fabrication of novel MOF-based sensor (JUK-2-MWCNTs-AuNPs/GCE) prepared by a simple one-step drop casting of a multicomponent suspension on the surface of a GCE. Our investigations proved that the JUK-2 MOF plays a crucial role in the unique performance of the developed sensor. It perfectly binds MWCNTs and AuNPs, ensures a very high reproducibility and accuracy of performed measurements, and guarantees a favorable synergistic effect of the nanomaterials used. The coexistence of proton and electron conductivity in the tested nanocomposite, which acts as a mixed ion-electron conductor (MIEC), is most likely responsible for an excellent electrocatalytic activity of the proposed sensor towards CIT oxidation with a wide linear range, low detection limit, high selectivity and good reproducibility. It is noteworthy that at all stages of the developed method, including JUK-2 synthesis and sensor fabrication, only environmental friendly reagents and solvents were used. Moreover, the analysis was carried out in a neutral environment, which makes the proposed method fully compliant with the principles of GAC. Finally, the satisfactory results obtained for CIT determination in all tested samples (tablets, water samples, biological material) clearly demonstrate the potential of the fabricated JUK-2-MWCNTs-AuNPs/GCE sensor for practical applications.

## Supplementary Information

ESM 1 (DOCX 1183 kb)

## Data Availability

Not applicable.
