# Supplementary material for: A voltammetric sensor based on mixed proton-electron conducting composite including metal-organic framework JUK-2 for determination of citalopram
Source: Mikrochim Acta. 2021 May 11;188(6):184. doi: 10.1007/s00604-021-04835-9 (PMC8113198; doi:10.1007/s00604-021-04835-9)
Supplement: Supplementary file 1 — (DOCX 1183 kb) [file 604_2021_4835_MOESM1_ESM.docx]

**Supplementary Material**

**Microchimica Acta**

**A voltammetric sensor based on mixed proton-electron conducting
composite including metal-organic framework JUK-2 for determination of citalopram**

Maria Madej^1,*^, Dariusz Matoga^2^, Klaudia Skaźnik^1^, Radosław Porada^3^, Bogusław Baś^3^, Jolanta Kochana^1,*^

**^1^** Jagiellonian University, Faculty of Chemistry, Department of Analytical Chemistry, Gronostajowa 2, 30-387, Kraków, Poland

**^2^** Jagiellonian University, Faculty of Chemistry, Department of Inorganic Chemistry, Gronostajowa 2, 30-387, Kraków, Poland

^3^ AGH University of Science and Technology, Faculty of Materials and Ceramics, Department of Analytical Chemistry, A. Mickiewicza 30, 30-059, Kraków, Poland

* **Corresponding author e-mail:** marysia.madej@doctoral.uj.edu.pl (https://orcid.org/0000-0002-7979-2922)

**
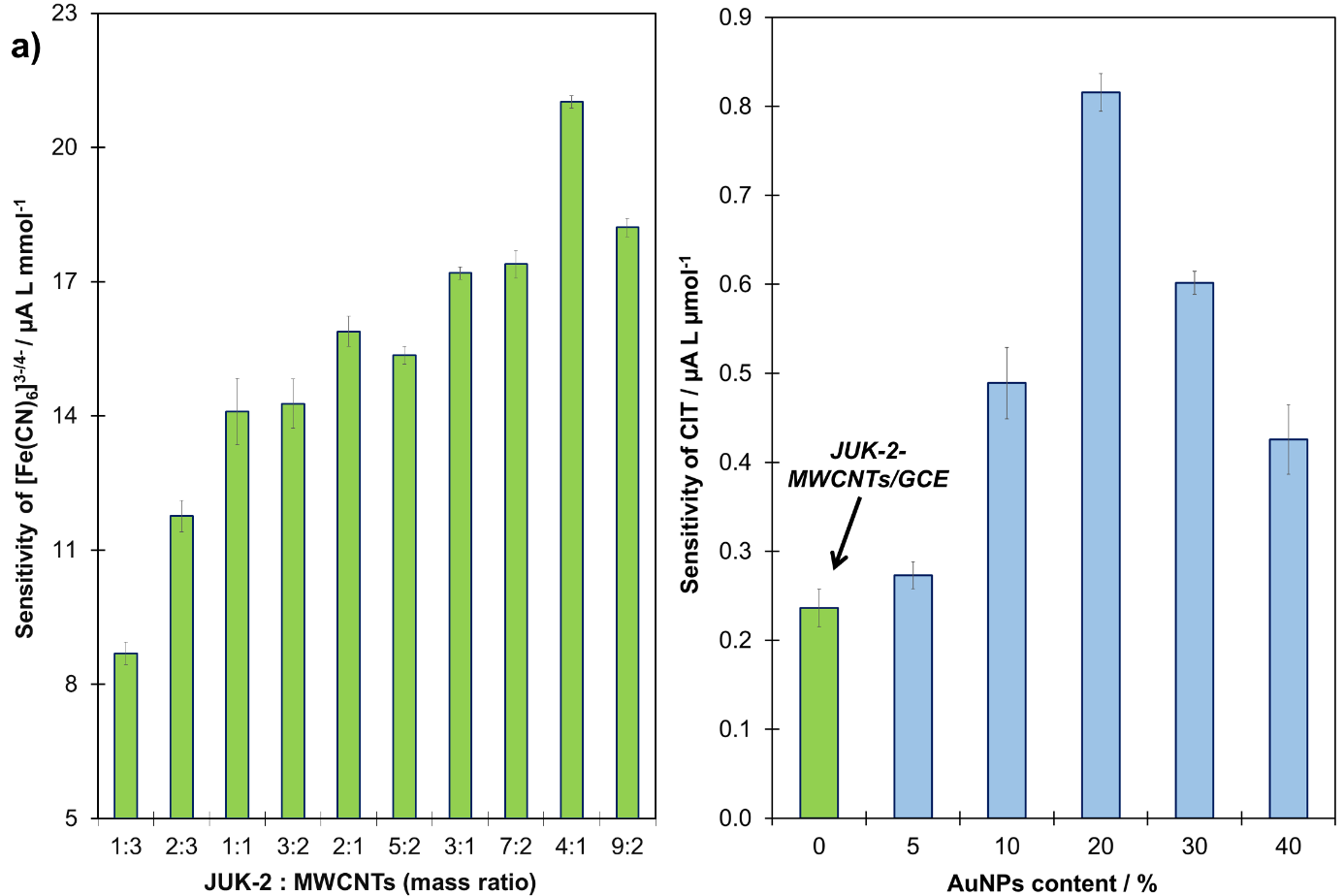
**

**Figure S1.** (a) Influence of JUK-2 and MWCNTs mass ratio used for sensor fabrication on the sensitivity obtained in CV measurements performed in 0.1 mol L^-1^ KCl containing 0.2 – 1.0 mmol L^-1^ [Fe(CN)_6_]^3-/4-^ (scan rate 0.05 V s^-1^; n = 3); (b) Impact of AuNPs content on the sensitivity achieved in CV measurements conducted in PBS (pH 7, 0.1 mol L^-1^) containing 10 – 30 µmol L^-1^ of CIT (scan rate 0.1 V s^-1^; E_acc_ = 0 mV, t_acc_ = 60 s).


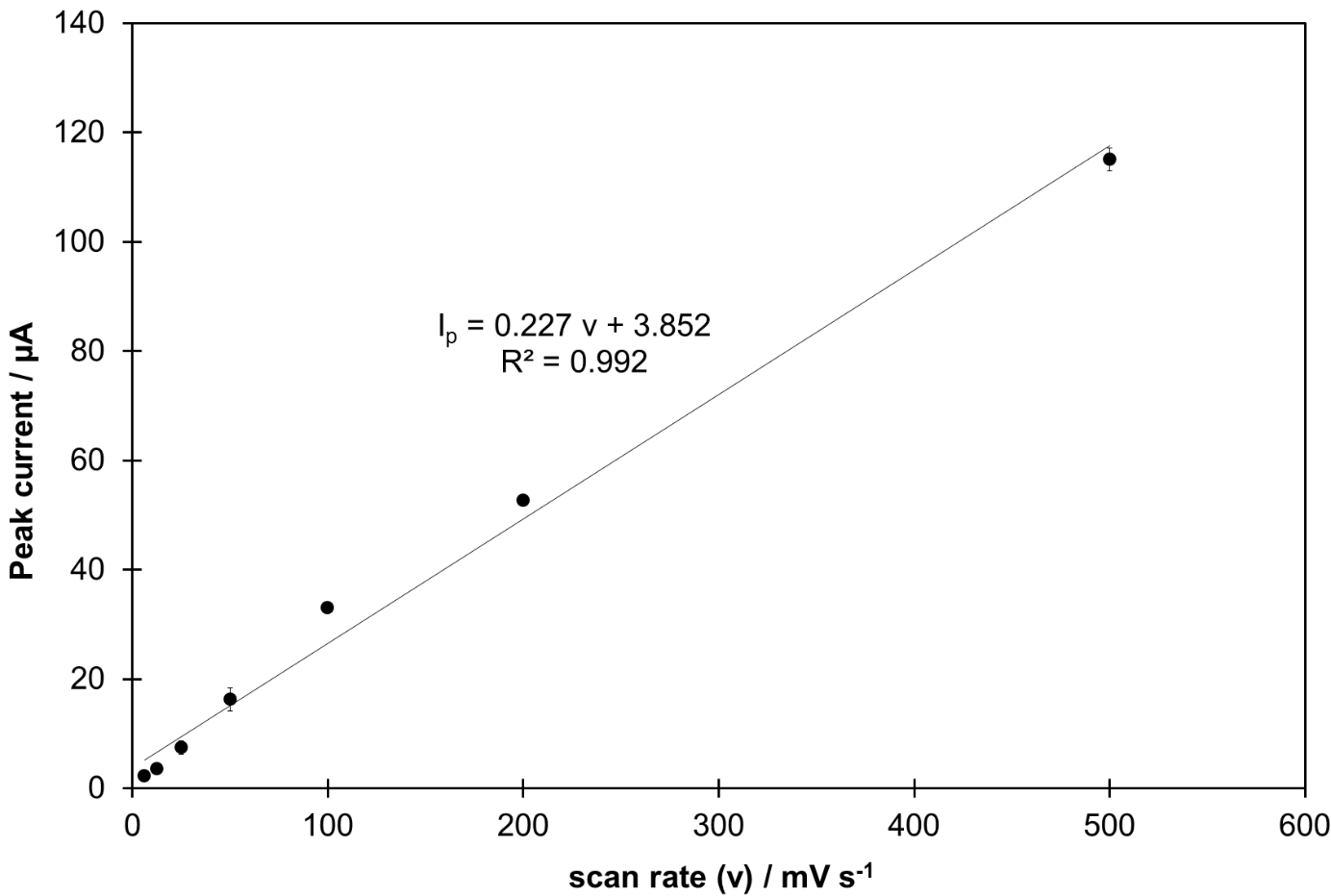


**Figure S2.** Dependence between the oxidation peak current and the scan rate obtained from CV measurements performed in PBS solution (pH 7, 0.1 mol L^-1^) containing 30 µmol L^-1^ of CIT (n = 3). (E_acc_ = 0 mV; t_acc_ = 60 s).


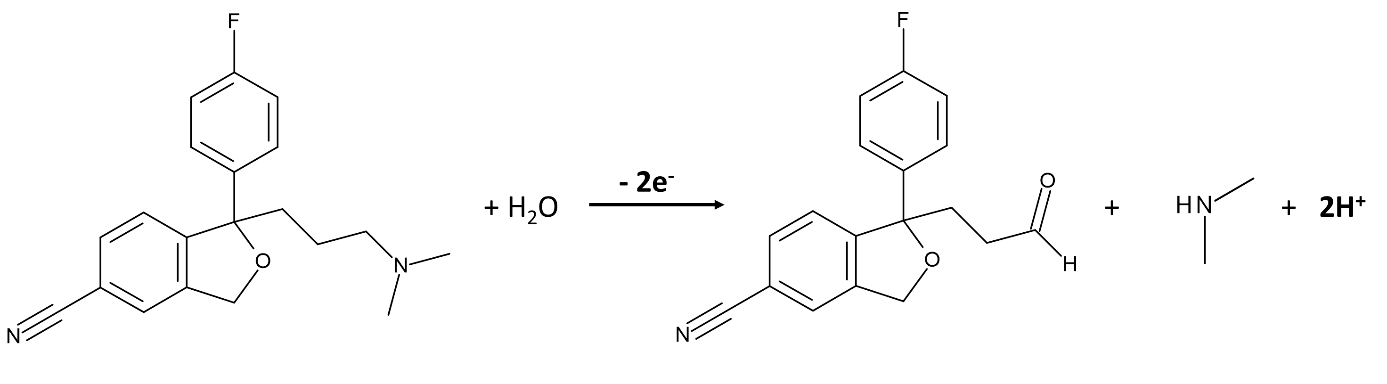


**Figure S3.** Proposed mechanism for CIT electrooxidation at JUK-2-MWCNTs-AuNPs/GCE.

***Optimization of SCV parameters***

The electrochemical preconcentration is an important stage of voltammetric measurements, especially for compounds showing a tendency for adsorption on an electrode surface. For this reason, the effect of accumulation time (t_acc_) and potential (E_acc_) of working electrode on the registered peak current were evaluated. Over the tested range of t_acc_ (*see Table S1*), the rapid growth of signal with increasing accumulation time up to 180 s was observed. For longer time, only slight increase was noted, probably due to a saturation of the electrode surface (Fig. S4a). Thus, accumulation time of 180 s was selected, as it combines good sensitivity and relatively short time of analysis. The effect of accumulation potential on the recorded signal was examined over the potential range of -200 to 600 mV. Obtained results (Fig. S4b) clearly shows that the maximum peak current is obtained for E_acc_ equal to 0 mV. Consequently, this value was chosen for subsequent measurements. The optimized parameters for CIT determination using SCV technique are summarized in Table S1.


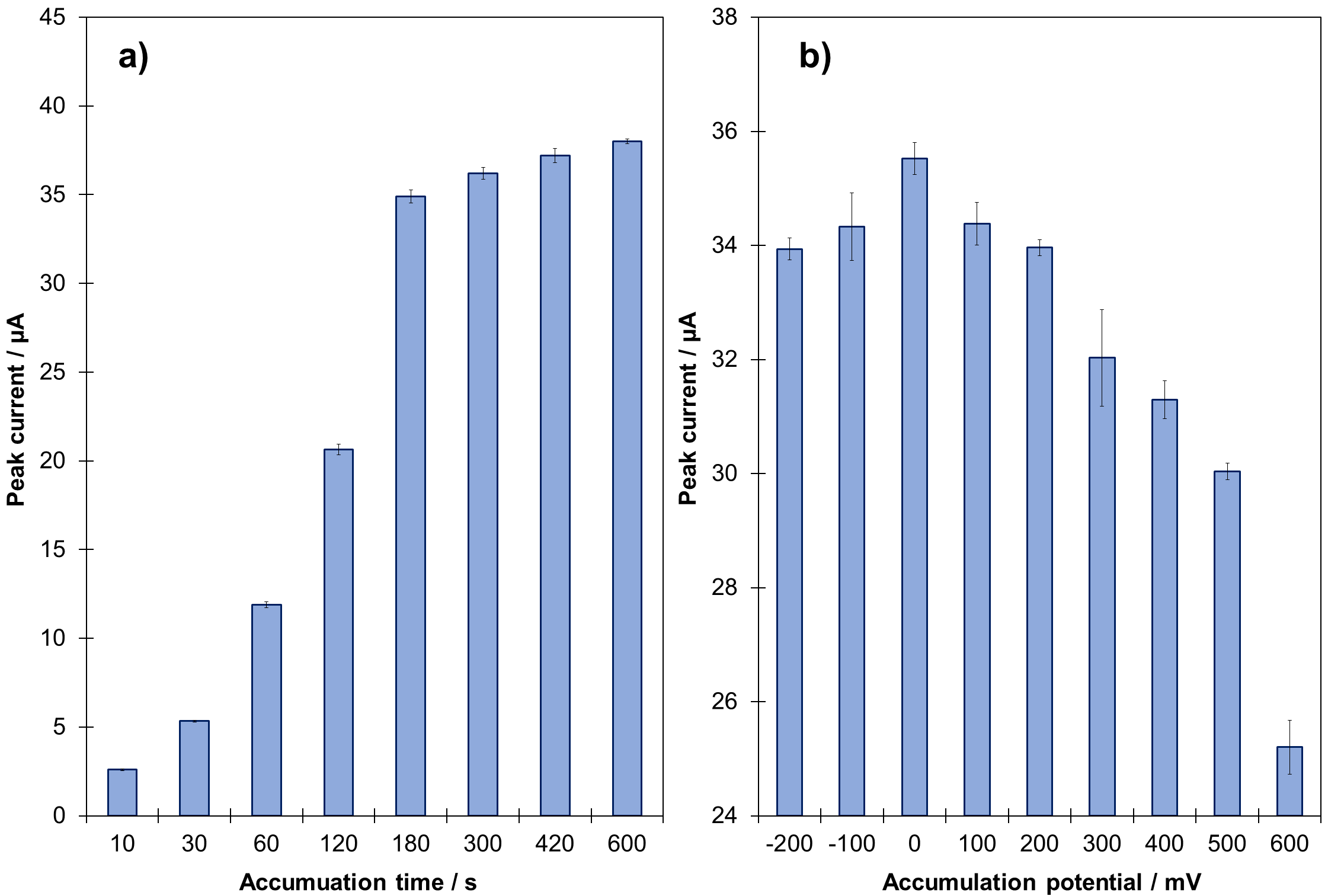


**Figure S4.** Dependence of the accumulation time with E_acc_ = 0 mV (a), and the accumulation potential with t_acc_ equal to 3 min (b) on the oxidation peak current recorded in PBS solution (pH 7; 0.1 mol L^−1^) containing 10 µmol L^−1^ of CIT using SCV technique (E_s_ = 8 mV; t_s_ = 40 ms; n = 3).

**Table. S1** Optimum values for tested SCV parameters.

| Parameter | Tested range | Chosen value |
| --- | --- | --- |
| Potential step (E_s_) | 1 – 8 mV | 8 mV |
| Step width (t_s_) | 16 – 640 ms | 80 ms |
| Accumulation time (t_acc_) | 10 – 600 s | 180 s |
| Accumulation potential (E_acc_) | -200 – 600 mV | 0 mV |


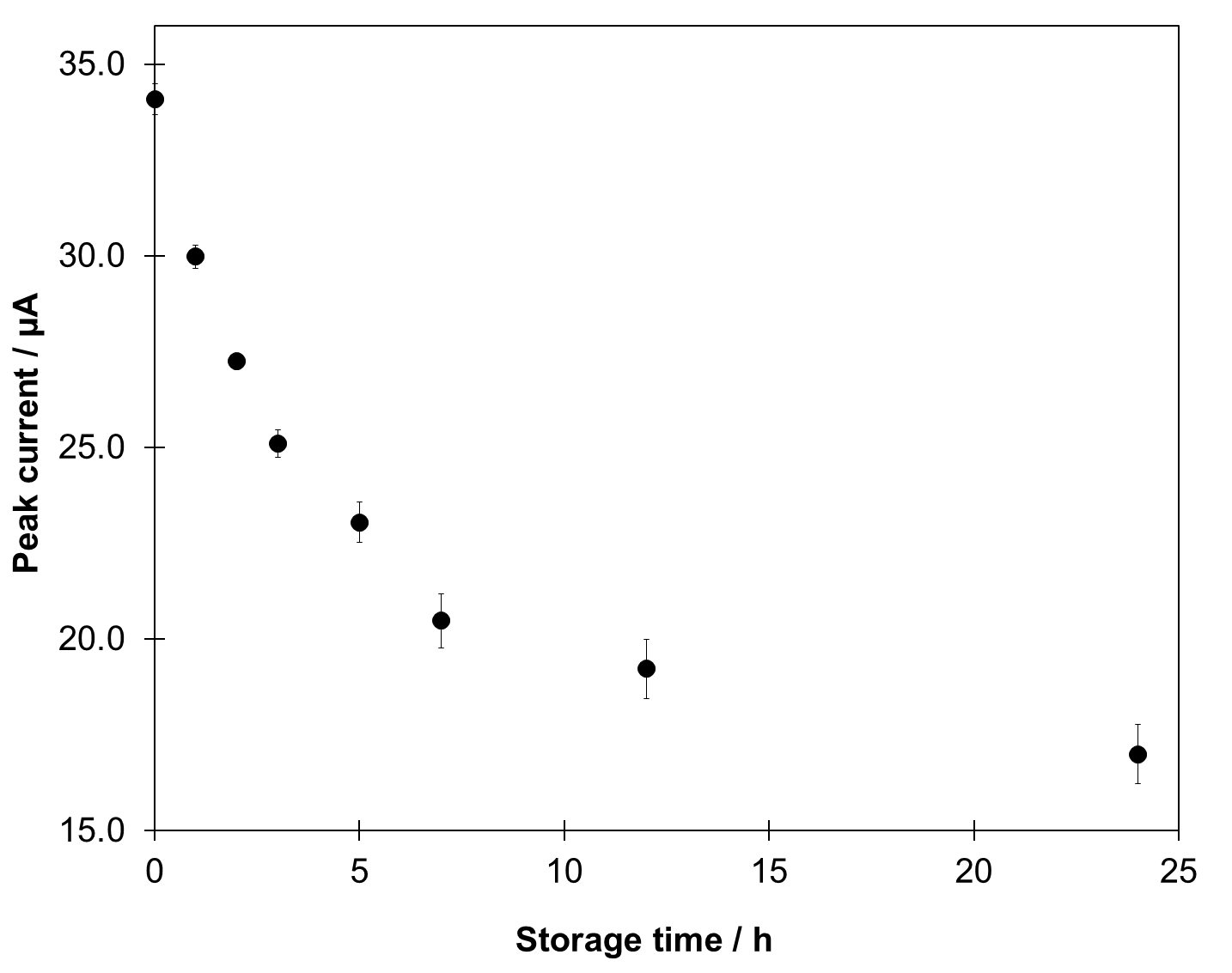


**Figure S5.** Influence of the sensor storage time on the recorded peak current in PBS solution (pH 7; 0.1 mol L^−1^) containing 10 µmol L^−1^ of CIT (SCV parameters: E_s_ = 8 mV; t_s_ = 40 ms, E_acc_ = 0 mV, t_acc_ = 180 s); zero value refers to the measurement carried out immediately after solvent evaporation; (n = 3).


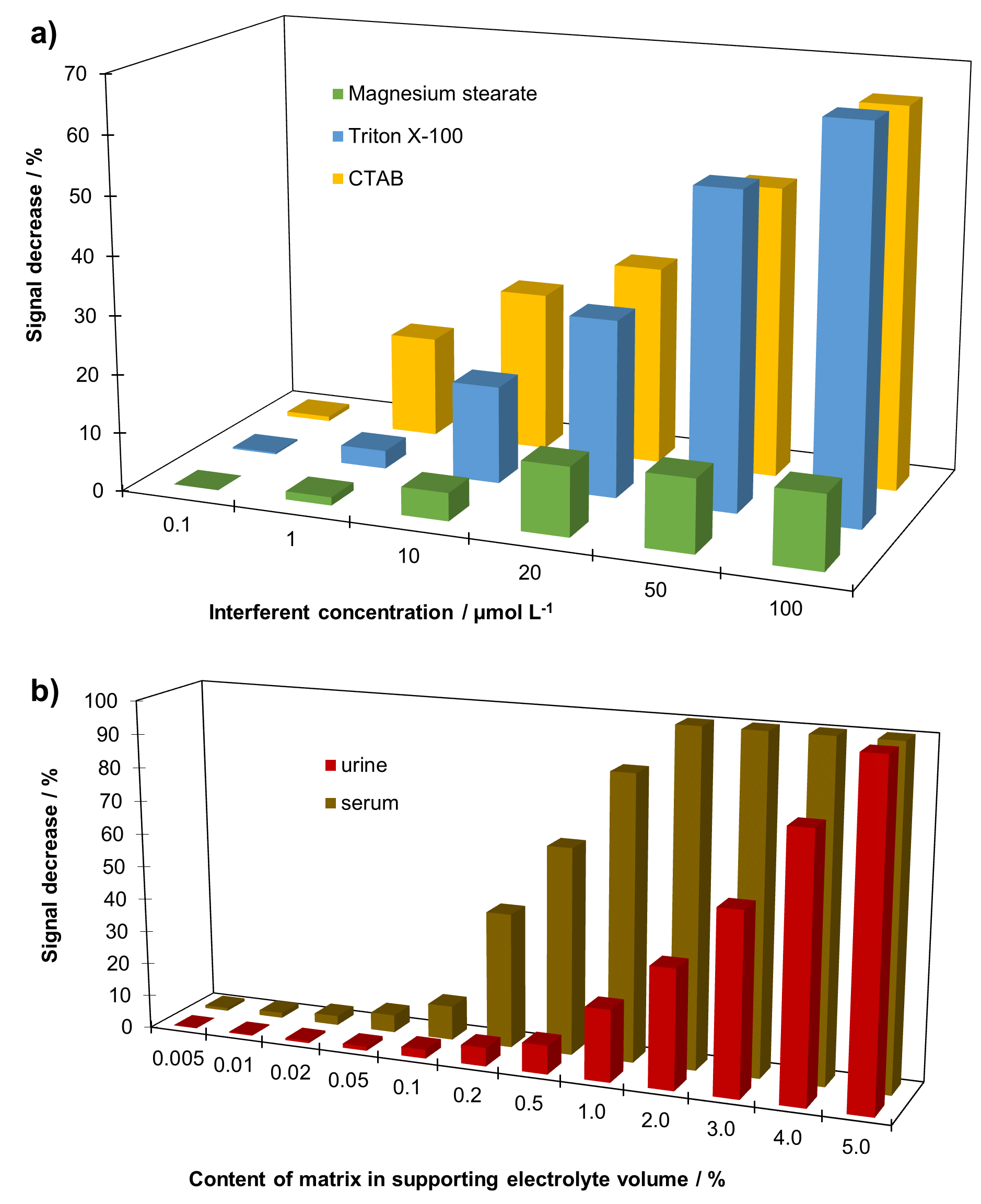


**Figure S6.** Signal changes caused by the presence of magnesium stearate (green), Triton X-100 (blue) and CTAB (yellow) (a) and by the occurrence of synthetic biological matrix (b) urine (red), blood serum (brown) in PBS (pH 7; 0.1 mol L^-1^) containing 10 µmol L^-1^ of CIT. SCV parameters: E_s_ = 8 mV; t_s_ = 80 ms, E_acc_ = 0 mV, t_acc_ = 180 s.

**Table S2.** Verification of interference effects of different inorganic and organic compounds on the oxidation peak current recorded for 10 µmol L^-1^ concentration of CIT.

| Tested interferent | Signal change (*%*) caused by the presence of an interferent in concentration / *µmol L^-1^* * | | | | | |
| --- | --- | --- | --- | --- | --- | --- |
|  | **0.1** | **1.0** | **10** | **20** | **50** | **100** |
| Ascorbic acid | -0.1 | -0.6 | -1.3 | -2.3 | -3.0 | -3.2 |
| Glucose | -0.4 | -0.9 | -2.8 | -2.6 | -3.1 | -2.1 |
| Lactose | -0.2 | -0.1 | -0.1 | -0.2 | -0.3 | -1.6 |
| TiO_2_ | -1.3 | -2.5 | -2.8 | -3.7 | -3.6 | -3.4 |
| Talc | -1.6 | -0.8 | -1.6 | -3.0 | -3.9 | -4.6 |
| Starch | 0.6 | -2.3 | -2.3 | -1.9 | -3.3 | -3.5 |
| Triton X-100 | -0.3 | -3.1 | **-16.6** | **-30.2** | **-53.7** | **-66.2** |
| SDS | -0.2 | -1.3 | -1.3 | -1.0 | -1.2 | -0.2 |
| CTAB | -0.8 | **-17.3** | **-27.3** | **-33.8** | **-49.5** | **-64.8** |
| Magnesium stearate | 0.2 | -1.4 | -4.8 | **-11.8** | **-12.4** | **-12.6** |

* negative value refers to the decrease in the citalopram oxidation peak current recorded in the presence of an interferent, whereas a positive value indicates an increase in the obtained signal.
